# Supplementary material for: Impact of a multicomponent physical exercise program on intrinsic capacity in community-dwelling older adults
Source: PeerJ. 2025 Mar 12;13:e19017. doi: 10.7717/peerj.19017 (PMC11910150; doi:10.7717/peerj.19017)
Supplement: Supplemental Information 4 [file peerj-13-19017-s004.docx]

**APPENDIX II – PROTOCOL FOR COLLECTING PHYSICAL VARIABLES**

**Weight**

The body weight of the elderly participants will be measured using a Plenna® portable digital scale. They will be given instructions in advance regarding the use of light clothing, and will be asked to step without shoes and with empty pockets onto the center of the scale base. The body should be upright, feet parallel and slightly apart, arms at the sides of the body and gaze straight ahead. The weight will be recorded in kilograms (kg). ^1^

**Stature**

Height will be measured using a Cescorf® portable stadiometer. The participant will be placed barefoot, standing, and facing away from the stadiometer with feet parallel, arms at sides and palms facing the body, head aligned and looking forward. The stadiometer will be positioned at the highest point of the head, compressing the hair enough to obtain the correct height measurement in m/cm. ^1^

**BMI**

Body mass index (BMI) will be calculated by dividing body mass by height squared (kg/m2) and classified according to Lipschitz (1994), in which underweight (<22 kg/m2), eutrophic (22-27 kg/m2) and overweight (≥27 kg/m2). ^2^

**Current body fat percentage, muscle mass percentage, visceral fat, basal metabolic rate and body age.**

Body composition will be assessed using a body control scale (bioimpedance scale), Omron ® model HBF-514C. Initially, the scale is configured with the participant's personal data (sex, age and height) and the participant is instructed to step onto the platform, barefoot, and place their feet on the electrodes, holding the display platform. The elderly person will then extend their arms outstretched at a 90º angle to their body and the scale will begin to calculate body composition. When the measurement is complete, the values for weight, BMI, body fat percentage, skeletal muscle percentage, basal metabolism, body age and visceral fat level will be displayed. The cutoff points adopted for muscle mass (MM) will be those recommended by EWGSOP2, in which low appendicular MM is considered <20 kg for men and <15 kg for women and appendicular ME/height² <7.0 kg/m² for men and <5.5 kg/m² for women . ^3^

**Hand grip strength**

To measure muscle strength, we will use the handgrip test using a Jamar® hydraulic dynamometer. The measurement will be obtained with the elderly person sitting with their elbows flexed at 90º and their forearm in a neutral position. We will ask the elderly person to pull the dynamometer handle as hard as possible for six seconds with their dominant hand and then relax. The command to perform and finish the task will be given out loud and three consecutive measurements will be taken, with a one-minute interval between them. Afterwards, the best result of the attempts will be selected. The measurement will be measured in kilograms/force (kgf) and adjusted for age and gender. The cutoff points adopted are those recommended by the EWGSOP2 < 27 kg for men and <16 kg for women . ^3^

**Lower limb strength**

The strength of the lower limbs of the body, necessary for tasks such as climbing stairs, walking, getting up from a chair or car will be assessed by the *30-Second Chair* Stand Test , of Rikli & Jones (1999) Functional Physical Fitness Test Protocol *.* ^4^ To perform the test, participants will be instructed to sit in the middle of a chair (with a backrest, without arms and approximately 43 cm high), with their backs straight, feet apart and resting on the floor. The elderly person will also be asked to cross their upper limbs at the level of the wrists and against the chest.

The instructor signals the start of the test and the participant must rise to the maximum extension (vertical position) and return to the initial seated position. The participant is encouraged to perform the maximum number of repetitions in a 30-second interval. The evaluator may make verbal or gestural calls to correct the performance of the activity, and at the end of the time counts the number of repetitions performed correctly. The reference values can be seen below:

|  | **Number of repetitions** | | | | | | |
| --- | --- | --- | --- | --- | --- | --- | --- |
| Age | 60-64 | 65 to 69 | 70 to 74 | 75 to 79 | 80 to 84 | 85 to 89 | 90 to 94 |
| Women | 15 | 15 | 14 | 13 | 12 | 11 | 9 |
| Men | 17 | 16 | 15 | 14 | 13 | 11 | 9 |

Source: Rikli and Jones, 1999, 2013. ^4.5^

**Functional Mobility**

To assess functional mobility, we will apply the *Timed Up and Go Test* (TUG), which measures, in seconds, the time it takes for a person to stand up from a standard chair (43 cm from the floor, without armrests), walk a distance of three meters and sit back down in the same chair. To perform the test, the elderly person will be asked to sit in the chair with their back against the back of the chair, with their knees flexed at a 90º angle. The test will be evaluated using the *Baiobit system* , which is a system that accurately quantifies the patient's motor function through a scientifically validated system . The test is considered normal when the time taken to complete the test is less than 10 seconds; between 10 and 19 seconds, the elderly person is at moderate risk of falling, and if the time is greater than 20 seconds, they are classified as having a high risk of falling. ^6^

**Perimeters (abdominal, thighs and calves).**

Abdominal and waist circumference will be measured with the participant in an upright position, with clothing pulled away from the abdominal region, and the researcher will position the tape measure around the waist at the midpoint between the lower edge of the last rib and the iliac crest, and the measurement will be recorded in cm. The parameters indicative of central obesity are: > 102 for men and > 88 cm for women, however, there are still no specific cutoff points for the elderly population. ^7^

The midfemoral circumference (MCC) is measured with the elderly person in an upright position, with their legs apart and their body weight resting lightly on their left leg. The measuring tape is positioned at the inguinal crease and, from the inguinal crease, the tape is extended to the upper edge of the patella and the midpoint of the thigh is marked for measurement. The measurement is recorded in centimeters.

Finally, to measure calf circumference, we will use an inelastic tape with the elderly person in an upright position, feet apart, with legs loose, without resting on the floor, in the area of the largest diameter of the calf. The cutoff points for CP vary according to the literature; for the present study, we will use the cutoff point of <31 cm for men and women according to other references. ^3,8^

**Blood pressure**

Blood pressure (BP) will be measured using an Omron® digital blood pressure monitor. Three measurements will be taken, one after five minutes of rest and the following at two-minute intervals; the final measurement will correspond to the average of the three measurements. The 2020 Brazilian Guidelines for Arterial Hypertension will be used as parameters ^.^

| **Blood pressure classification from 18 years of age** | | | |
| --- | --- | --- | --- |
| **Classification** | **SBP (mmHg)** |  | **DBP (mmHg)** |
| **Great PA** | < 120 | and | <80 |
| **Normal BP** | 120-129 | and/or | 80-84 |
| **Prehypertension** | 130-139 | and/or | 85-89 |
| **HA stage I** | 140-159 | and/or | 90-99 |
| **HA stage II** | 160-179 | and/or | 100-109 |
| **HA stage III** | ≥ 180 | and/or | ≥110 |

HA: arterial hypertension; BP: blood pressure; SBP: systolic blood pressure; DBP: diastolic blood pressure.

Source: Brazilian Hypertension Guidelines (2020)

**Heart Rate**

Heart rate measurement will be performed at the time of blood pressure measurement using an Omron® digital blood pressure measuring device.

**Flexibility**

The flexibility of the lower limbs, including the posterior thigh and lower body muscles, will be assessed by the Chair Sit and Reach Test . Functional Physical Fitness Test Protocol Rikli & Jones (1999). ^4^ For the procedure, participants will be positioned seated at the end of a chair approximately 43 cm high. The chair is placed against a wall to maintain stability and safety.

Participants will be instructed to bend one knee and extend the other toward the thigh with the heel on the floor and the ankle flexed (approximately 90º). With the knee extended, but not overextended, the elderly person should lean forward, with the spine erect and the head aligned with the spine, and should try to touch the toes by sliding the hands, one on top of the other, with the tips of the middle fingers, on the extended leg. This position should be held for two seconds and the subject will be reminded to exhale as he or she leans forward, avoiding quick and forced movements and never exceeding his or her pain threshold. The participant should try it twice, followed by the application of the test.

Once the task has been completed, the evaluator uses a 45 cm ruler to measure the distance (cm) to the toes (minimum result) or the distance (cm) reached beyond the toes (maximum result). The middle of the big toe, at the end of the shoe, represents the zero point. Both values found will be recorded to the nearest 1 cm and the best result will be used to evaluate performance. The reference values can be seen below:

|  | **Centimeters** | | | | | | |
| --- | --- | --- | --- | --- | --- | --- | --- |
| Age | 60-64 | 65 to 69 | 70 to 74 | 75 to 79 | 80 to 84 | 85 to 89 | 90 to 94 |
| Women | -0.5 - +5.0 | -0.5 - +4.5 | -1.0 - +4.0 | -1.5 - +3.5 | -2.0 - +3.0 | -2.5 - +2.5 | -4.5 - +1.0 |
| Men | -2.5 -+  4.0 | -3.0- +3.0 | -3.5 - +2.5 | -4.0 -+2.0 | -5.5 - +1.5 | -5.5- +0.5 | -6.5 - -0.5 |

Source: Rikli and Jones, 1999, 2013. ^4.5^

**Balance**

Static balance will be assessed by the Single-Leg Balance Test, which assesses the elderly person's ability to maintain a single-leg position on the supporting leg for a maximum of 30 seconds. This study will consider the following times: <20 seconds (risk), 21-30 seconds (normal), and >31 seconds (excellent), according to the data proposed in the literature. ^10^

**REFERENCES**

1. Brazil. Ministry of Health. Guidelines for the collection and analysis of anthropometric data in health services. Available at: <https://bvsms.saude.gov.br/bvs/publicacoes/orientacoes_coleta_analise_dados_antropometricos.pdf>. Accessed on August 5, 2023.
2. Lipschitz, DA. Screening for nutritional status in the elderly. Primary Care: Clinics in Office Practice [Internet] 1994 [Accessed 8 August 2023]; 21(1): 55-67. Available at: <https://doi.org/10.1016/S0095-4543(21)00452-8>.
3. Cruz-Jentoft AJ, Bahat G, Bauer J, Boirie Y, Bruyère O, Cederholm T, et al. Sarcopenia: revised European consensus on definition and diagnosis. Age Aging [Internet] 2019 [Accessed August 9, 2023];48(1):16–31. Available at: <https://doi.org/10.1093/ageing/afz046>.
4. Rikli RE, Jones CJ. Development and validation of a functional fitness test for community-residing older adults. J Aging Phys Act [Internet] 1999 [Accessed 10 January 2023];7(2):129–61. Available from: <https://doi.org/10.1123/japa.7.2.129>.
5. Rikli RE, Jones CJ. Development and validation of criterion-referenced clinically relevant fitness standards for maintaining physical independence in later years. Gerontologist [Internet] 2013 [Accessed January 10, 2023]; 53 (2): 255-67. Available at: <https://doi.org/10.1093/geront/gns071>.
6. Podsiadlo D, Richardson S. The timed "Up & Go": a test of basic functional mobility for frail elderly persons. J Am Geriatr Soc [Internet] 1991 [Accessed 13 August 2023];39(2):142–8. Available at: <https://doi.org/10.1111/j.1532-5415.1991.tb01616.x>.
7. Mayoral LP, Andrade GM, Mayoral EP, Huerta TH, Canseco SP, Rodal Canales FJ et al. Obesity subtypes, related biomarkers & heterogeneity. Indian J Med Res [Internet] 2020 [Accessed 13 August 2023];151(1):11-21. Available from: doi: 10.4103/ijmr.IJMR_1768_17.
8. Pinheiro PA, da Silva Coqueiro R, Carneiro JAO, Correia TML, Pereira R, Fernandes MH. Anthropometric indicators as screening tools for sarcopenia in older adult women. Enferm Clin [Internet] 2020 [Accessed 15 August 2023]; S1130-8621(19):30041–30045. Available from: <https://doi.org/10.1016/j.enfcli.2018.12.010>.
9. Barroso WKS, Rodrigues CIS, Bortolotto LA, Mota-Gomes MA, Brandão AA, Feitosa ADM, Machado CA, et al. Brazilian Guidelines for Hypertension – 2020. Arq. Bras. Cardiol [Internet]. 2021 [Accessed on August 15, 2023];116(3):516-658. Available from: <https://doi.org/10.36660/abc.20201238>.
10. Bohannon RW. One-legged balance test times. Percept Mot Skills [Internet] 1994 [Accessed 16 August 2023];78(3 Pt 1):801-2. Available at: <https://doi.org/10.1177/003151259407800322>.
