# Supplementary material for: Impact of a multicomponent physical exercise program on intrinsic capacity in community-dwelling older adults
Source: PeerJ. 2025 Mar 12;13:e19017. doi: 10.7717/peerj.19017 (PMC11910150; doi:10.7717/peerj.19017)
Supplement: Supplemental Information 5 [file peerj-13-19017-s005.docx]

**APPENDIX III- INTERVENTION DETAILS**

The following table summarizes the main components worked by different types of exercise, highlighting muscular strength, muscular power, cardiorespiratory fitness, flexibility, balance, coordination and the neuromuscular component.

| **Modality** | **Muscle Strength** | **Muscle Power** | **Cardiorespiratory Fitness** | **Flexibility** | **Balance** | **Coordination** | **Neuromuscular Component** |
| --- | --- | --- | --- | --- | --- | --- | --- |
| **Bodybuilding** | **✓** | **✓** |  |  | **✓** | **✓** | **✓** |
| **Aerobic Exercises** |  |  | **✓** |  | **✓** | **✓** |  |
| **Circle Dance** |  |  | **✓** | **✓** | **✓** | **✓** | **✓** |
| **Chinese Healing Gymnastics** |  |  | **✓** | **✓** | **✓** | **✓** | **✓** |
| **Pilates Matwork** | **✓** |  |  | **✓** | **✓** | **✓** | **✓** |
| **Gerontomotricity** |  |  | **✓** | **✓** | **✓** | **✓** | **✓** |
| **Pedestrianism** |  |  | **✓** |  | **✓** | **✓** |  |
| **Maintenance gymnastics** | **✓** |  | **✓** | **✓** | **✓** | **✓** | **✓** |
| **Water activities** | **✓** |  | **✓** |  | **✓** | **✓** | **✓** |
| **Nordic Walking** |  | **✓** | **✓** |  | **✓** | **✓** | **✓** |

Table 1: Components worked in each exercise modality.

| **MODALITY** | **DESCRIPTION** |
| --- | --- |
| **Bodybuilding** | Bodybuilding is a form of training with therapeutic, aesthetic, leisure, rehabilitation and health promotion purposes.  In PIAFI, bodybuilding is divided into a beginners' sheet and an intermediate sheet, each with 26 workouts. The first four workouts are designed to help the elderly person adapt and are 1x15 (one series of fifteen repetitions) for each exercise; from the fifth to the thirteenth workout, there are 2x12/15 (two series with twelve or fifteen repetitions each); from the fourteenth to the twenty-sixth workout, there are 3x10 (three series with ten repetitions). After the 26th workout, the intermediate sheet begins and continues at 3x10 (three series with ten repetitions) for each exercise with a higher level of intensity.   \| Session \| Volume \| \| \| \| --- \| --- \| --- \| --- \| \| Series \| Repetitions \| Rest \| \| 1st to 4th training \| 1 \| 15 \| - \| \| 5th to 13th training \| 2 \| 12 or 15 \| 30 to 45 seconds \| \| 14th to 26th training \| 3 \| 10 \| 30 to 45 seconds \| \| After the 27th training \| 3 \| 10 \| 30 to 45 seconds \|   **beginners ' sheet consists of 26 sessions with 12 exercises that alternate between upper and lower limbs, which are:**  Upper limbs:  Single-arm cross row  Reverse flyer  Extension Chair  Triceps Pulley  Dumbbell Bicep Curl  Seated Wide Row* Straight Bar  Reverse flyer  Multifunctional high row  Adductor chair  Triceps dumbbell forehead (flat bench press)  Dumbbell or Direct Hammer Curl  with bar on the multifunctional  Lower limbs  Squat with or without holding the backrest  Extension Chair  Abductor and Adductor Chair  Plantar flexion  Pelvic elevation (Gluteus bascula)  Back Flexion  Dumbbell Sumo Squat  Unilateral shin flexor.  Backrest or floor support (circular choice)  Seated plantar flexion (soleus)  device  Abdomen  Dynamic or static supra abdominal  Unilateral paravertebral with 4 supports or decubitus spine extension (circular choice)  **Afterwards, the intermediate and maintenance form is started, consisting of 26 sessions of 12 exercises, normally with the exercises already provided in the previous form, although new exercises may be added. They are:**  Upper Limbs  Low Row  Bench Press  Reverse Flyer  Alternating Front and Side Raises  Multifunctional Triceps  Multifunctional Biceps  High Pull  Shoulders on the machine  Triceps Forehead  Bicep Curl  Lower limbs  Knee Extension  Knee Flexion  Abductor/Adductor Chair  Gemini Sitting on the Machine  Pelvic elevation  Dorsal Flexion of the ankle  Abdomen  Supra Abdominal  Note _1_ : Before starting the exercises, stretches are performed: lower back, back of legs, iliopsoas and glutes. |
| **Aerobic Exercises** | These are long-lasting, continuous exercises of light and/or moderate intensity, which are closely related to the cardiovascular and respiratory systems.  Part 1 - Warm-up (10 minutes):  General Warm-up: Warm-up series that involve all muscle groups, such as:  March in place.  Hip abduction (raising the knees).  Rotation of the arms.  Gentle stretching of neck, shoulders, arms, legs and ankles.  Part 2 - Main Cardiovascular (30 minutes):  Cardiovascular activity preferred by the elderly (bicycle, treadmill, rowing machine, elliptical). Maintaining a rhythm that raises the heart rate. The goal is to maintain a moderate and constant heart rate. Use heart rate monitors to monitor heart rate during activity.  The heart rate for cardiovascular training is prescribed in the physical assessment of the elderly person and recorded on the cardiovascular training form. To calculate the training frequency, we use the formula proposed by Karyonen: FCT = % (HRMax – HRRep) + HRRep.  Part 3 - Cool Down and Stretch (10 minutes):  Cool Down Activity (5 minutes): Gradually decrease intensity.  Stretching (5 minutes): Perform gentle stretches for the legs, arms, back and shoulders to relax the muscles |
| **Circle Dance** | The practice of Circular Dance is an instrument for personal development, building self-awareness and creating a sense of connection and collectivity through choreographies with different focuses: acting on physiological, locomotor, vitality and meditation demands.  Classes will last from 45 minutes to 1 hour and 30 minutes, with music and choreography, focusing on the connection between the participants in the Circle. Circle Dance promotes balance between the physical, mental and spiritual bodies. The practice invites participants to be present and express their emotions through dance in a deliberate and positive way. |
| **Chinese Healing Gymnastics** | Chinese Healing Gymnastics is a set of body practices, derived from ancient forms of training for health promotion, based on the principles of Traditional Chinese Medicine. It is an integrated system of body practices aimed initially at the prophylaxis and treatment of disorders of the locomotor system and later at neurological disorders .  Classes are held in groups and last from 45 minutes to 1 hour and 30 minutes. During these classes, 8, 12 and 18 exercises are performed with 2 or 3 series, divided as follows:  1 - Anterior Liang Gong or Pain Protocol  6 exercises for the neck and shoulders;  6 exercises for the trunk;  6 exercises for the lower limbs.  2- Liang Gong Posterior  6 exercises for joint pain in the extremities;  6 exercises for tendosynovitis;  6 exercises for internal organ dysfunctions.  3 - Wai Tan kung - Gymnastics of the Immortals, consisting of 12 muscle relaxation exercises.  4 - Liang Gong Xi Ba Fa  18 exercises for the prevention and treatment of chronic bronchitis and heart disease.  5 - Chi Kung Tai Chi  18 exercises to boost immunity.  6 - Ba Duan Din - Silk Embroidery Exercises - The 8 Health Exercises. |
| **Pilates Matwork** | Pilates is a method of physical and mental conditioning that aims to restore health and promote an increase in the quality of life of those who practice it and thus enjoy its numerous benefits. The modality in the PIAFI project will last 45 minutes and will be organized as follows: Initial moment of the class (5-minute warm-up): pre-Pilates and Pilates exercises with the aim of bringing awareness and focus to the class, mobilizing the main joints and warming up the body in preparation for the exercise routine. Main moment (35 minutes): development of the main sequence of exercises that will be developed according to the objective and level of the practitioners involved. Final moment (5 minutes): the finishing exercises must be precise and stimulate awareness of the exercises performed in the main part, the perception of better axial stretching, the relaxation of muscles that do not require too much tension, the transfer of the exercises performed to other daily activities.  Compositions of 8 to 10 exercises with 6 to 10 repetitions are performed (except for Hundread which is 100 repetitions):  Cat Streach  Ab pre  Shoulder bridge pre  One leg circle  Side leg lift Right  Breast Stroke  Side leg lift Left  Shell Streach  Spine Twist  Spine Streach  Hundread  Immediately afterwards, stretching is performed. |
| **Gerontomotricity** | The practice is carried out through primary tasks with aerobic, balance and coordination physical components, combined with a cognitive skill such as attention, memory, information processing speed and executive functions.  Part 1 - Warm-up (5 minutes):  Cognitive activity (e.g. memory game)  Part 2 - Coordination and Cognition Exercises (10 minutes):  -Walk and Count (2 minutes):  Walk while counting from 1 to 20, alternating between walking and stopping after each even number.  -Ball Juggling (2 minutes):  Throw and catch light balls while walking in circles.  -Play Ball and Recite (2 minutes):  Throw a soft ball between participants while reciting poetry or naming objects around them.  -Backward Walk (2 minutes):  Walk backwards for a short distance.  -Crossword Game (2 minutes):  Solve simple crossword puzzles while walking.  Part 3 - Balance and Coordination Exercises (10 minutes):  -Walking on Lines (2 minutes):  Walk in a straight line, trying to keep your feet exactly on a line on the ground.  -Leg Lifts (2 minutes):  Lift one leg at a time while maintaining balance.  -Spin and Catch (2 minutes):  Turn your body in a circle and pick up objects on the floor.  -Crossing Obstacles (2 minutes):  Place obstacles on the ground and walk through them.  -Arm Swing (2 minutes):  Swing your arms back and forth while walking.  Part 4 - Stretching and Relaxation (5 minutes):  Stretching of upper and lower limbs.  Deep breathing and relaxation. |
| **Pedestrianism** | The form of hiking or walking refers to walking routes, generally in natural and rural environments, which in many countries are marked with internationally known marks and codes.  The sessions will last between 30 minutes and 1 hour and 30 minutes, with guidance from the teacher who will monitor parameters such as adequate gait pattern, breathing, target heart rate, and perceived exertion. The class will be preceded by a warm-up of the large and small joints, with emphasis on the lower limbs, and stretching of the large muscle groups and those most used during the practice. The activity will be carried out on open trails in nature with identified signage markings.  Part 1 - Warm-up (0-5 minutes):  Start with a slow walk.  Warming up the joints, with emphasis on the lower limbs, and stretching the large muscle groups.  Part 2 - Moderate Walk (5-15 minutes):  Moderate pace.  Part 3 - Brisk Walking (15-30 minutes):  Increase in pace.  Part 4 - Moderate Walk (30-35 minutes):  Speed reduction.  Part 5 - Hill Climb (35-40 minutes):  Inclusion of hill climbing, if available.  Part 6 - Cooling Down (40-45 minutes):  Decreased intensity for slow and stationary walking. |
| **Maintenance Gymnastics** | Maintenance gymnastics is an activity that aims to work on physical skills in a global way, especially: muscular strength, muscular power, cardiovascular resistance and flexibility.  Initial moment (mobility exercises) – 10 minutes  Shoulder rotation  Knee raises  ankle rotation  Neck stretch  Pelvic rotation  Main Part (30 minutes)  -Exercises for Upper Limbs  Push-ups (3 sets of 10 repetitions).  Wall push-ups (3 sets of 12 reps).  Dumbbell lateral raises (3 sets of 12 reps).  Triceps dips on a sturdy chair (3 sets of 10 reps).  -Exercises for Lower Limbs  Squats (3 sets of 12 reps).  Lunges (3 sets of 12 repetitions on each leg).  Standing calf raises (3 sets of 15 reps).  Step-ups on a sturdy bench (3 sets of 10 reps on each leg): Step up and down from a sturdy bench.  -Core Exercises  Abdominal crunches (2 sets of 15 reps)  Final Moment (10 minutes):  Leg stretching.  Stretching arms and shoulders.  Relaxation and deep breathing. |
| **Water activities** | The aquatic activities modality serves a target audience with low mobility, functionality and conditioning, such as elderly people in pre-frailty or frailty conditions who need to develop conditioning and coordination skills through the aquatic environment to enable these skills that are diminished or affected by circumstances of chronic diseases in more severe conditions such as Osteoporosis with a history of fragility fractures, Parkinson's, Fibromyalgia, Arthrosis, and more severe lower back pain.  The aquatic activities program includes series of exercises performed in a heated pool at 32°C to 34°C to overcome and reduce fear of engaging in exercise. The modality will use a package of 10 sessions for the practitioner based on functional assessments. The proposal is to serve groups of 5 to 6 elderly people accompanied by an instructor who will be teaching the class in the water in order to promote safety and better care and monitoring of the exercises. The sessions will be 45 minutes long.  Initial Part  Warm-up (5 minutes):  Walking or moving around the pool.  Breathing Exercises (5 minutes)  Deep, slow breathing.  Main Part  The main part can be carried out in the form of a group class, circuit or routes.  Hydrobike (15 minutes):  Aquatic pedaling in sets of 3-4 minutes with 1 minute rest.  Resistance Exercises (15 minutes) - 2-3 minute sets with 1 minute rest  Lateral arm raises with weights.  Leg flexion and extension with resistance.  Side kicks in the water.  Flexion and extension of the ankles in water.  Water aerobics exercises (10 minutes) - 2-3 minute sets with 1 minute break.  Hip abduction in water.  Raising your knees in the water.  Flexion and extension of the shoulder joints.  Rotation of the arms in the water.  Final Part  Cool Down Walk (5 minutes):  Slower walk in the pool.  Relaxation and Stretching (5 minutes): |
| **Nordic Walking** | Nordic Walking classes will last 1 hour, including warm-up, walking and ending with stretching/cooling down. Various activities may also be included, such as: circuit activities with poles, strength training and recreational activities in sessions held individually or in groups.  Initial Moment (10 minutes)  Preparing the Poles (5 minutes): Checking the appropriate height of the poles for each participant (umbilical scar)  Warm-up (5 minutes): Light walking to prepare your muscles.  Main Part (30 minutes):  Walking with Poles.  Use of sticks to propel steps.  Keep your elbows bent at about a 90 degree angle.  Using arm movement to help with walking.  Final Moment (5 minutes):  Cool Down Walk (2 minutes): Slow down the pace and continue walking in a more relaxed manner.  Stretching (3 minutes): Perform gentle stretches for major muscle groups, including legs, arms and back. |

**Table 2.** Details of the intervention consisting of multicomponent exercises.


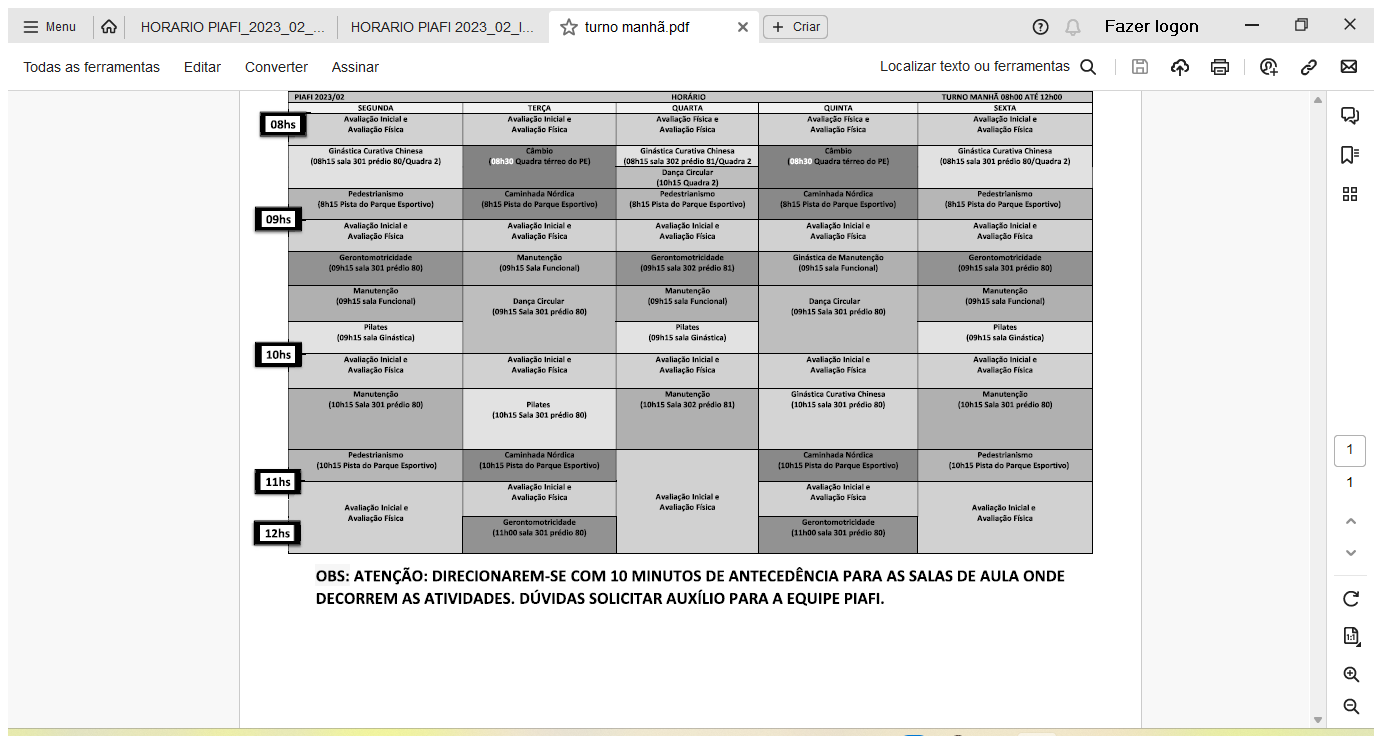


Table 3. Timetable of multicomponent exercises for the morning shift.


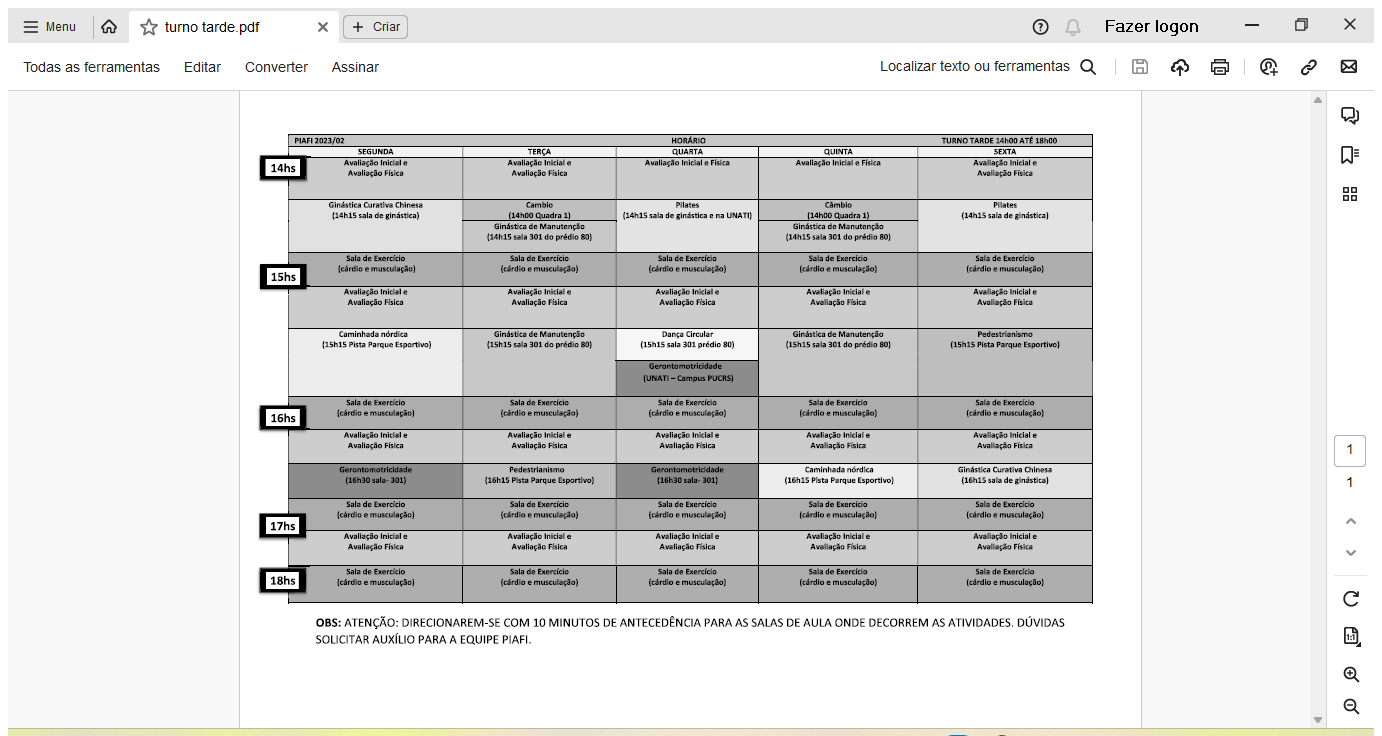


Table 4. Timetable for multicomponent exercises in the afternoon shift.


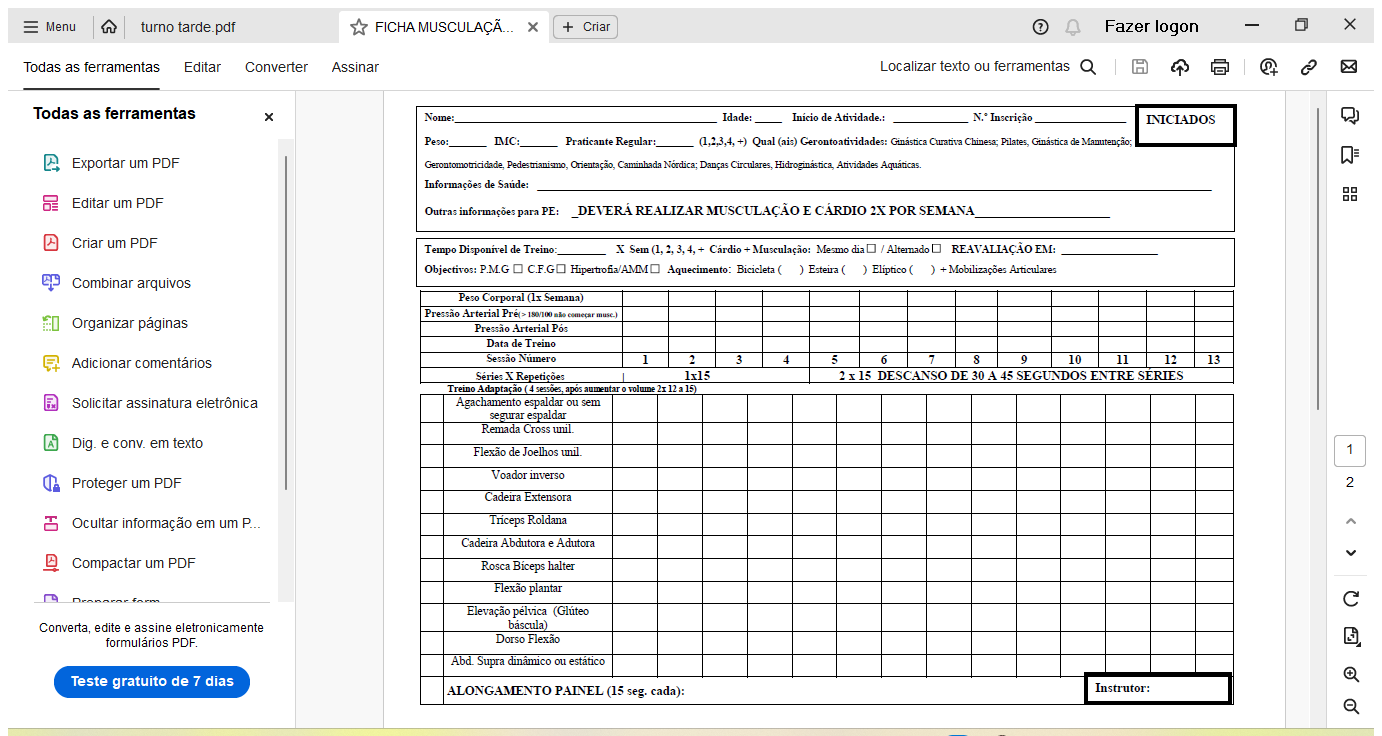


Figure 2. Bodybuilding sheet for beginners in front.


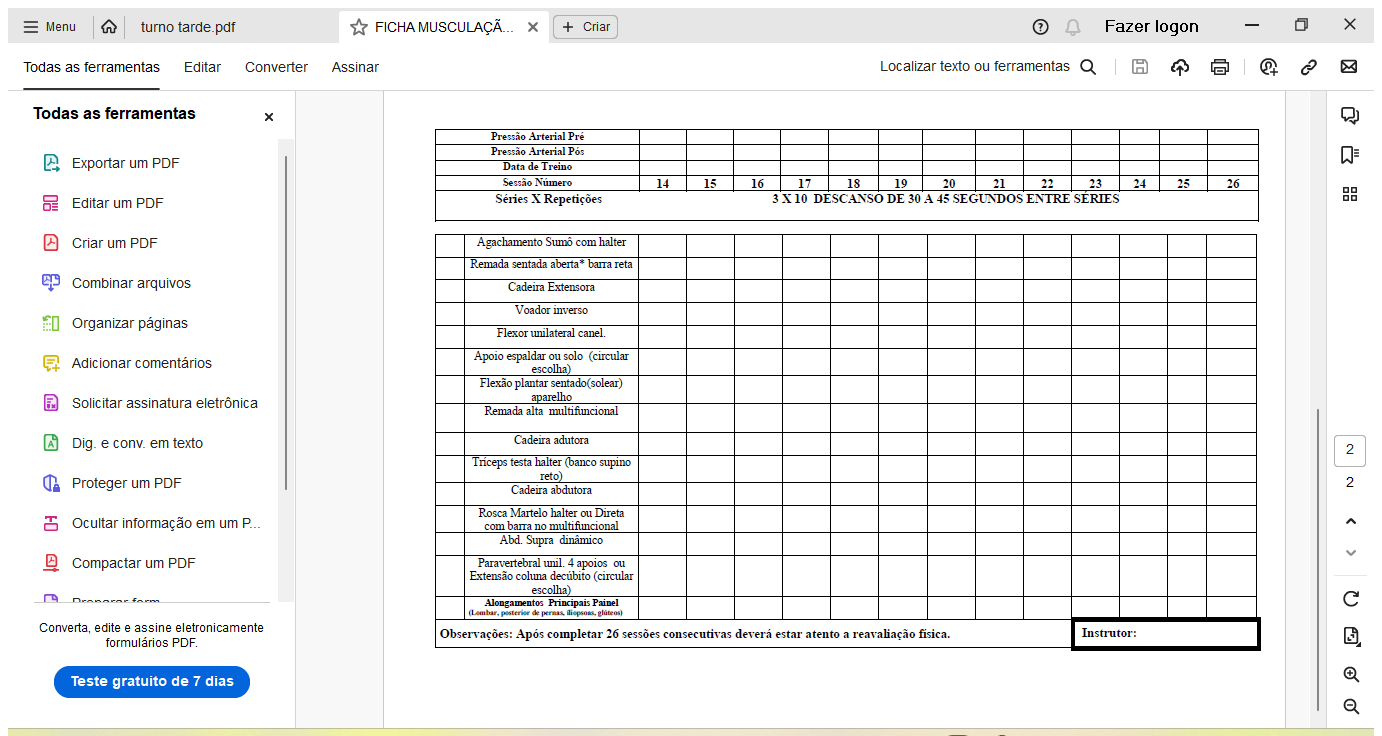


Figure 3. Beginners' bodybuilding form, back side.


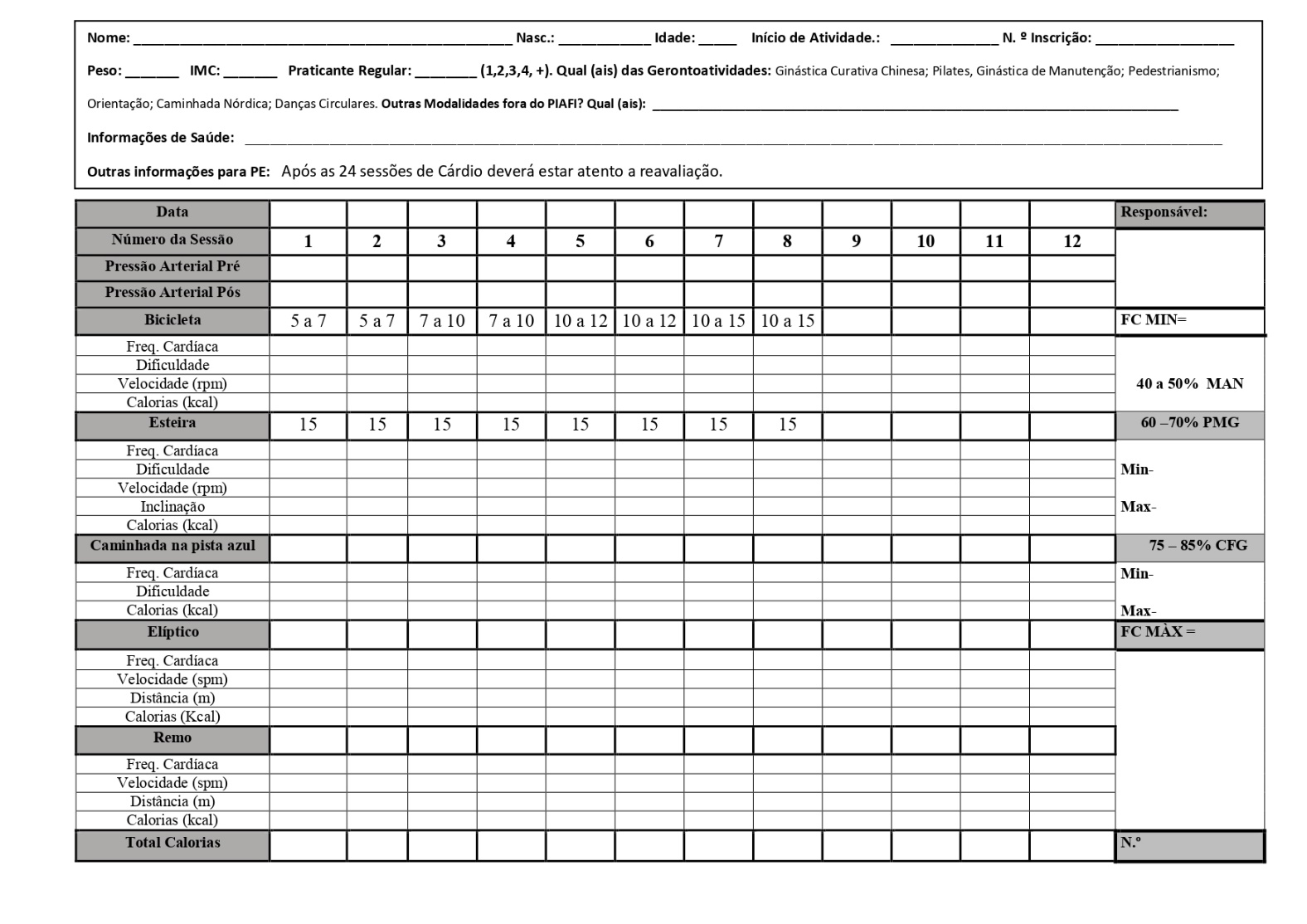


Figure 4. Cardiovascular training sheet front.


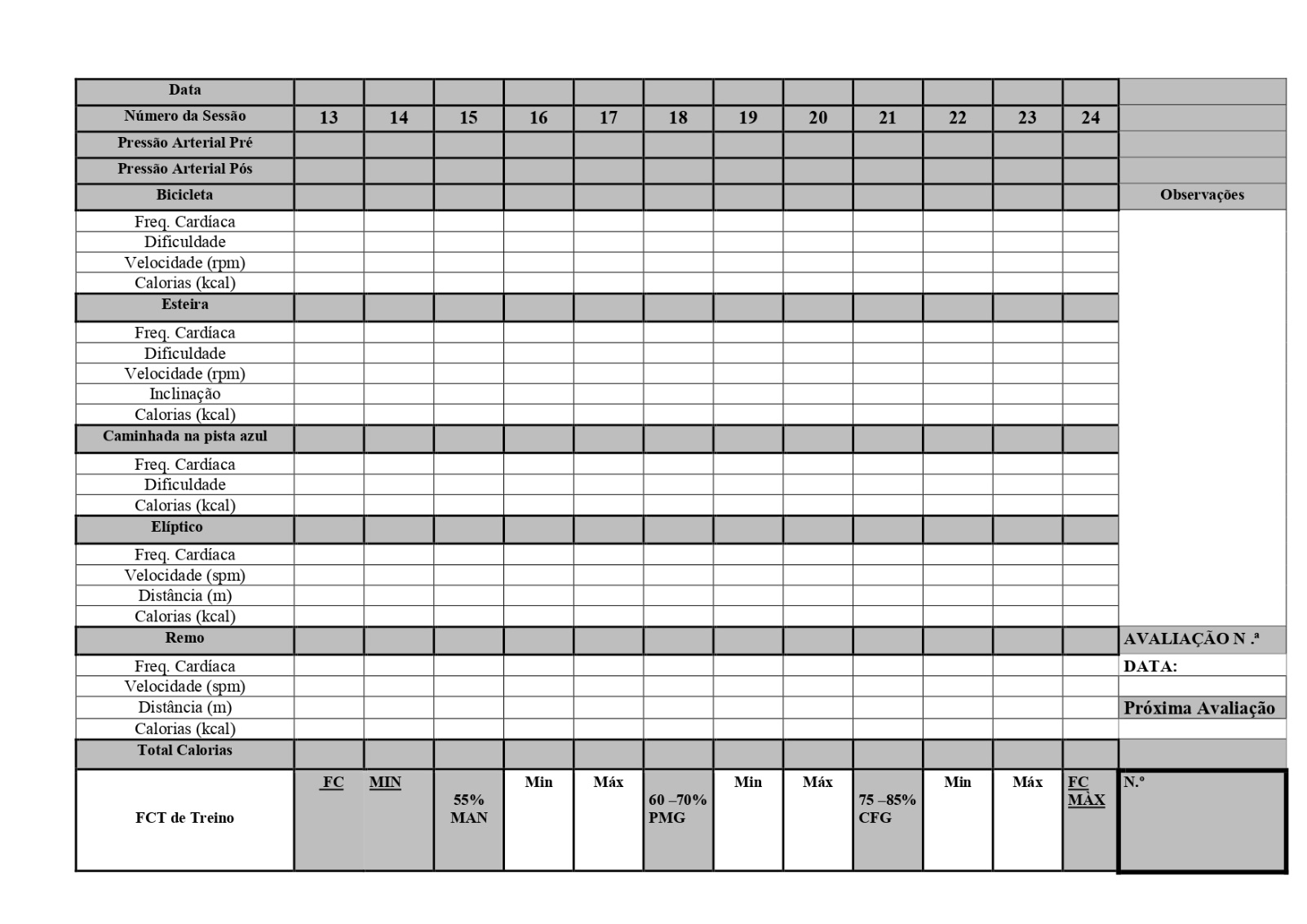


Figure 5. Cardiovascular training sheet back.
